# Perceived safety in human–cobot interaction for fixed-path and real-time motion planning algorithms

**DOI:** 10.1038/s41598-022-24622-7

**Published:** 2022-11-28

**Authors:** Inara Tusseyeva, Artemiy Oleinikov, Anara Sandygulova, Matteo Rubagotti

**Affiliations:** Department of Robotics and Mechatronics, School of Engineering and Digital Sciences, Nazarbayev University, 010000 Astana, Kazakhstan

**Keywords:** Electrical and electronic engineering, Mechanical engineering, Information technology

## Abstract

This study investigates how different motion planning algorithms, implemented on a collaborative robot (cobot), are perceived by 48 human subjects. The four implemented algorithms ensure human safety based on the concept of *speed and separation monitoring*, but differ based on the following characteristics: (a) the cobot motion happens either along a fixed path or with a trajectory that is continuously planned in real time via nonlinear model predictive control, to increase cobot productivity; (b) the cobot speed is further reduced—or not—in real time based on heart rate measurements, to increase perceived safety. We conclude that (1) using a fixed path—compared to real-time motion planning—may reduce productivity and, at least when heart rate measurements are not used to modify the cobot speed, increases perceived safety; (2) reducing cobot speed based on heart rate measurements reduces productivity but does not improve perceived safety; (3) perceived safety is positively affected by habituation during the experiment, and unaffected by previous experience.

## Introduction

Traditional industrial manipulators are typically large and heavy, and designed for high-accuracy, high-speed and high-volume production. Their workspace in a factory is usually separated from that of human operators by cages, to avoid harmful impacts^1^. To allow workspace sharing with humans, recent years have seen an increase in the use of collaborative robots—often simply named *cobots*—which, compared to traditional industrial manipulators, are smaller, lighter and move with lower speeds, thus allowing for physical human–robot interaction (pHRI) without physical barriers^2,3^.

An important role in pHRI is played by safety standards. Their aim is to guarantee that, if any injuries are sustained by human operators, they are mild contusions in the worst-case scenario^4^. The ISO/TS 15066 standard^5^ defines, among others, two main strategies for autonomous robot motion when the workspace is shared with a human, namely *speed and separation monitoring (SSM)* and *power and force limiting (PFL)*. In the case of SSM^6,7^, the robot speed is increased proportionally to the distance with the human, to ensure that a collision never occurs while the robot is in motion. Instead, PFL is aimed at guaranteeing an upper bound on the impact force associated with any possible collision, by limiting the robot speed irrespective of the distance with the human^8,9^. These strategies are typically associated to a fixed path, in which the robot speed is modulated so as to guarantee the required condition. To enhance productivity with respect to fixed-path strategies, different frameworks have been proposed to replan the robot motion in real time, while still guaranteeing ISO/TS 15066 safety standards^10–13^.

In addition to investigating safety, several works in the past decade have focused on assessing aspects related to the perception of safety of human operators when interacting with a cobot^14–18^. These works constitute a subset of a wider research effort aimed at studying perceived safety while interacting with different types of robots^19^. Though still in its infancy, this field of research is of practical relevance, as the continuous interaction with a robot can cause mental strain and negative cognitive-emotional reactions in human operators^20,21^. In the field of pHRI, perceived safety was defined by Bartneck et al.^22^ as “the user’s perception of the level of danger when interacting with a robot, and the user’s level of comfort during the interaction”, and is tightly related to other concepts, such as stress, anxiety and fear. In particular, perceived safety is connected with one of the components of stress, i.e. the appraisal of threat, defined as “the perception that one might experience harm”^18^. For a human interacting with a cobot, perceived safety and related aspects have been evaluated using different methods, such as physiological assessment^17,18^—consisting of measuring variables such as heart rate (HR) and Galvanic skin response (GSR) during experiments^23^—and questionnaires^14,16–18^.

The aim of this paper is to provide an assessment of perceived safety for cobots, when these are operated either via a fixed-path motion planning algorithm—in which the cobot speed is modulated via SSM, but its motion never deviates from a predetermined path—or via a real-time motion planning algorithm—in which the whole cobot motion, including its path, is redefined at each sampling instant also imposing SSM. Several papers have shown that perceived safety in pHRI depends on aspects such as robot speed, distance with the human, robot size and appearance, motion fluency and predictability^19^. A cobot operated via real-time motion planning exhibits a motion that is in general more difficult to predict than that generated by a fixed-path algorithm. Thus, one would expect that, even if real-time planning can enhance productivity, the price to pay for its use would be a worsened perception of safety by the human operator.

Specifically, we implemented the fixed-path SSM algorithm with continuous speed modulation described by Marvel et al.^6^, and the real-time motion planning algorithm relying on nonlinear model predictive control (MPC) described by Oleinikov et al.^12^, on a Kinova Gen3 cobot. As it is known that speed is a factor that influences perceived safety, we also implemented variants of the two above-mentioned motion planning algorithms, in which the cobot speed was decreased in real time proportionally to the measured HR. We expected that these variants would reduce cobot productivity and increase perceived safety, and we were interested in determining the extent of these expected variations. To the best of our knowledge, real-time physiological feedback to modify the robot motion was used only in a limited number of works^24–27^ and never for cobots. In particular, in the paper by Rani et al.^24^, the so-called *anxiety index* was obtained in real time based on the cardiac response, the electrodermal activity and the electromyographic response of each human participant. A mobile robot explored the environment around the human, but immediately came closer to ask if any help was needed, in case the anxiety index exceeded a given threshold. Liu et al.^25,26^ proposed a robot-based basketball game in which the hoop was attached to the end effector of an industrial manipulator that moved with different speeds in different directions. The robot motion, and thus the difficulty of the game, was influenced in real time by the level of anxiety—estimated based on several sources of physiological measurements—of the human player. Finally, Kulic and Croft^27^ focused on safe motion planning algorithms for a traditional industrial manipulator in the presence of a human operator. The level of anxiety of the participant was assessed based on HR, GSR and corrugator muscle activity signals, and the robot speed was reduced accordingly as the assessed level of anxiety increased.

This paper will analyze the results of experiments with four motion planning algorithms: (1) fixed-path (FP), (2) fixed-path with HR-based speed modulation (FP-HR), (3) MPC, and (4) MPC with HR-based speed modulation (MPC-HR). Contrary to the work of Oleinikov et al.^12^, in which the presence of the human participants was only simulated, 48 different subjects participated in the experiments described in this paper. The position of several locations of the participants’ bodies, provided in real time to the cobot control algorithm, was acquired by an Optitrack optical motion capture system. Similarly to the work of Pollak et al.^18^, perceived safety was evaluated both through the assessment of physiological stress—via HR measured by an Empatica E4 wristband—and through questionnaires. The main contribution of our work is to assess how cobot productivity and perceived safety are affected by the characteristics of the specific algorithm used for motion planning. In particular, actual human safety for all algorithms is guaranteed via SSM, and the important features according to which the algorithms are compared are (a) the fact that the cobot motion happens on a fixed path or on a path that is continuously changed and (b) the absence or presence of mechanisms to further reduce the cobot speed based on HR monitoring. We also analyze the influence on cobot productivity and perceived safety of habituation during the experiments, and previous experience working with robots.

In “Materials and methods”, some important information is provided regarding how the bounds on the cobot speed are determined via SSM, the difference between the FP and the MPC algorithms, and how the cobot speed is modulated for the FP-HR and MPC-HR algorithms. The same section also provides a description of the research hypotheses, of experimental setup and experimental procedure, and of the measures employed to test the hypotheses. In “Results” and “Discussion”, we present the outcome of our statistical analysis and its discussion.

## Materials and methods

### Motion planning algorithms

The objective of the manipulator is to execute a sequence of point-to-point motions, each time steering the cobot configuration (vector of joint angles) $\varvec{\theta }\in {\mathbb {R}}^{n_\theta }$, where $n_\theta$ is a positive integer number representing the number of joints, to a given goal value. As a human operator is sharing the workspace with the cobot while executing an independent task, safety has to be ensured. This is achieved according to the above-mentioned SSM principle, which is explained in detail in the following.

#### Speed and separation monitoring

This section provides a description of the SSM algorithms based on the ISO/TS 15066 SSM criterion detailed in the paper by Marvel et al.^6^, and specifically described by Oleinikov et al.^12^. The SSM framework relies on the assumption that the speed of the human is never above a maximum value ${\bar{v}}_h$ determined by safety standards, equal to 2 m/s in our experiments. Under the worst-case condition in which human operator and robot—with the former moving at maximum speed—are moving towards each other, using SSM the robot can always come to a halt before the operator collides with it.

A conservative coverage of the space occupation of robot and human is obtained with a number of spheres, each centered at a specific time-varying location (e.g., at the right elbow of the human, or the center of the third robot link). In the considered case study, for example, we used $n_h=14$ spheres for the human and $n_r=7$ spheres for the robot. The centers coordinates—in a given fixed reference frame—and radii of the $n_h$ spheres on the human operator are referred to as ${\varvec{p}}_{h,j}\in {\mathbb {R}}^3$ and $R_{h,j}$, respectively, with $j=1,\ldots ,n_h$. The speeds of these sphere centers are assumed never to exceed the fixed upper bound ${\bar{v}}_h$. Similarly, centers coordinates—in the same reference frame used for the human—and radii of the $n_r$ spheres on the robot are indicated as ${\varvec{p}}_{r,i}\in {\mathbb {R}}^3$ and $R_{r,i}$, respectively, with $i=1,\ldots ,n_r$. The (scalar) speed of ${\varvec{p}}_{r,i}$ is referred to as $v_i$. The distances $d_{ih}$—between the $i$th robot sphere and the union of all human spheres—and $d_{rh}$—between the unions of all robot spheres and all human spheres—can be obtained, respectively, as

1 $$\begin{aligned} d_{ih}=\min _{j=1,\ldots , n_h}\left\{ d_{ij}-(R_{r,i}+R_{h,j})\right\} ,\quad d_{rh}=\min _{i=1,\ldots , n_r}d_{ih}, \end{aligned}$$

where $d_{ij}\triangleq \Vert {\varvec{p}}_{r,i}-{\varvec{p}}_{h,j}\Vert$ is the distance between the corresponding sphere centers. Negative values of $d_{ij}-(R_{r,i}+R_{h,j})$, and consequently of $d_{ih}$, indicate that a collision between human and robot spheres has occurred. The implementation of SSM is aimed at enforcing the following condition:

2 $$\begin{aligned} {\bar{v}}_h \left( T_{dr}+\frac{v_i}{{\bar{a}}_r}\right) +v_iT_{dr} + \frac{v^2_i}{2{\bar{a}}_{r}}+\varepsilon _s\le d_{ih},\ i=1,\ldots ,n_r, \end{aligned}$$

in which ${\bar{a}}_r$ represents the maximum deceleration—defined as a positive real number, and equal to 5 m/s$^2$ in our experiments—that can be imposed by the motors to the robot sphere centers, $T_{dr}$ is the detection (of the human position) and reaction time of the robot—equal to 100 ms, i.e., twice the sampling period of 50 ms—while $\varepsilon _s$ represents the precision of detection of the human position, obtained with the available sensors, equal to 4 mm. In (2), the term ${\bar{v}}_h \left( T_{dr}+v_i/{\bar{a}}_r\right)$ represents the maximum distance that the operator can travel before the robot detects it, reacts to it (i.e., calculates what control signal to provide to the motors), and finally comes to a halt; instead, the term $v_iT_{dr} + v^2_i/(2{\bar{a}}_{r})$ provides the distance that the *i*th robot sphere center covers during the same time interval. The sum of these two quantities, plus the human measurement precision $\varepsilon _s$, cannot be larger than the currently measured distance $d_{ih}$ between human and *i*th robot sphere center. If this holds for all robot spheres, then human–robot collisions with non-zero robot speed are impossible. The maximum allowable speed of each robot sphere center, which we name ${\bar{v}}_i$, can be obtained at each sampling time by solving the quadratic equation corresponding to inequality (2).

Given a nominal trajectory that the manipulator follows in the absence of the human operator, assume that, at any given time instant, the speed of the $i$th robot sphere center is equal to ${\hat{v}}_i$. The satisfaction of (2) for the FP algorithm is obtained by scaling all robot joint speeds of a quantity

3 $$\begin{aligned} c=\min \left( \min _{i=1,\ldots ,n_r}\frac{{\bar{v}}_i}{{\hat{v}}_i},1\right) , \end{aligned}$$

since the relationship between robot sphere center speeds and joint speeds is linear^28^. A continuous modulation of the robot speed will be employed in this paper as described above; however, it is important to remark that, in most industrial applications, the robot speed is changed based on thresholds on the human–robot distance $d_{rh}$, thus achieving a lower performance.

#### Fixed path versus real-time motion planning

The FP algorithm ensures safety, but the cobot productivity—inversely proportional to the average time needed for the cobot to complete each task—can be low if the human often obstructs the cobot path. As a possible solution, Oleinikov et al.^12^ proposed a real-time motion planning algorithm that determines the robot joint speeds based on a prediction of the robot motion given the current human operator pose. As already briefly mentioned in the introduction, the motion planning algorithm is implemented via MPC, solving at each sampling time—hence the term *real-time*—a nonlinear optimal control problem that minimizes a suitable cost function—accounting for quantities such as the distance of the robot configuration from its reference, and the joint speeds—while satisfying a number of constraints point-wise in time. These constraints include bounds on joint angles and speeds, avoidance of fixed obstacles, and a slightly more conservative version of the SSM constraint described in (2).

The details of this control law are outside the scope of this paper and not necessary to understand its contribution; the interested reader is referred to Oleinikov et al.^12^ for its detailed explanation. In order to provide a fair comparison, the cobot path for the FP algorithm is determined by running MPC without human presence; this ensures that, when the operator is not present, FP and MPC approximately generate the same robot motion, and have the same productivity.

**Figure 1:**
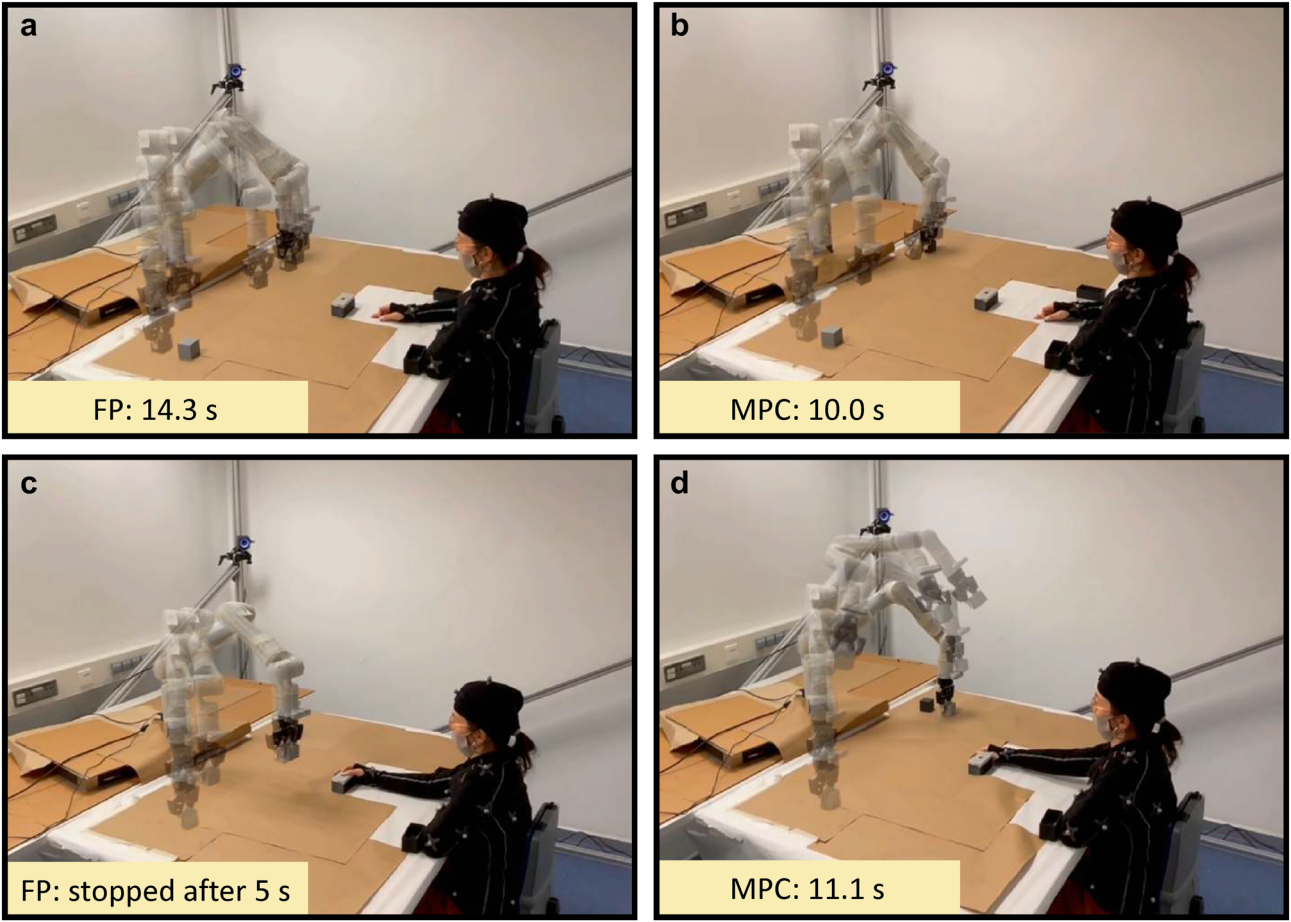
Cobot motion for the shown static positions of the human operator, with the shown numerical value indicating task completion time for the given pick-and-place task. As this figure only illustrates the difference between algorithms and is not directly related to the analyzed data, the shown human operator is not an actual participant of the experiments, but one of the authors of this paper.

For the reader’s convenience, we have shown the behavior of the two algorithms in Fig. 1, in which the cobot carries out a pick-and-place task, moving a cube from the left to the right of the operator, with the latter remaining still in the shown pose. The task completion time (for the pick-and-place task) is shown for each case in the figure. In Fig. 1a,b, the human was sufficiently close to influence the cobot motion: FP only slowed down the cobot along its pre-determined path, while MPC opted for moving it a bit farther from the human, thus being able to move faster according to the SSM principle, and achieving a lower task completion time. In Fig. 1c,d, with the human closer to the cobot, FP had to stop its motion after 5 s to avoid violating SSM; on the other hand, MPC planned a completely different path, increasing the height of the end effector during the motion, thus being able to keep a distance from the human that allowed the cobot to complete its task. As typically the human operator is constantly moving to carry out a given task, the robot motion never stops completely when using FP, but resumes once the human–robot distance increases again. This, however, can cause a significant reduction in robot productivity.

#### HR-based speed modulation

It is worth noticing that decreasing the robot speed proportionally to the distance with the human—which is exactly what happens when the SSM condition (2) is enforced—can have a benefit on perceived safety as well, as humans typically tend to feel safe if the robot speed decreases with distance^19^. However, as perceived safety is also proportional to robot speed regardless of the distance with the human^19^, it might be beneficial to further reduce it whenever the human operator feels uncomfortable. Thanks to the availability of low-cost sensors included in user-friendly equipment such as wristbands, monitoring the physiological parameters of the human operator is a concrete possibility in nowadays industrial environments. Specifically, the Empatica E4 wristband employed in our experiments provides measurements of HR and GSR with a sampling interval of 1 s. It is known that GSR can be considerably influenced by muscle contraction, and it is therefore difficult to use it during tasks that require a prolonged motion of the human^29^; indeed, preliminary experiments with our setup (with GSR measurements) confirmed that its value steadily increased as the operator carried out the assigned task, and its value could not be associated with the interaction between human and robot. As a consequence, we decided not to rely on GSR, but only on HR, similarly the work of Pollak et al.^18^.

In our opinion, the reduction of the cobot speed cannot directly rely on the measured HR value, as different subjects have different values of resting HR, typically varying between 60 and 100 beats per minute. Therefore, we decided to define our *perceived safety index* $\sigma$ based on the HR increase beyond a baseline level $HR_0$. The latter represents the average HR value that has to be measured for each subject before performing the task. To define $\sigma$, it is also necessary to determine for which value of the difference $HR-HR_0$ the subject can be considered sufficiently stressed or scared to justify a considerable decrease of the cobot speed; we refer to this value as $HR_\Delta$. Experiments to evaluate the range of HR variability due to stress and fear were conducted with human participants watching scary movies^30^ or pictures^31^, executing cognitive tasks^32^ and interacting with robots^33–35^. The study of Weistroffer et al.^34^, which studied the acceptability for human participants of the presence of robots in assembly lines, is the closest to our work. In it, a value of $HR_\Delta$ equal to 20 beats per minute (bpm) was estimated, and this value will be used in our paper for normalization purposes. Therefore, at each sampling instant, the value of $\sigma$ will be defined as

4 $$\begin{aligned} \sigma =\max \left\{ \frac{HR-HR_0}{HR_\Delta },0\right\} . \end{aligned}$$

The HR value is never expected to decrease below $HR_0$, but should that happen, we would obtain $\sigma =0$ according to (4). The value of $\sigma$ would typically vary between 0 and 1, with possible time intervals in which it would exceed 1. Notice that the perceived safety index is inversely proportional to perceived safety, but we preferred to maintain this terminology, as the concept of *perceived safety* is rather well established in the literature. The FP-HR and MPC-HR algorithms would be obtained by instantaneously redefining the value of ${\bar{v}}_i$ for each robot sphere center with a new value ${\bar{v}}_i'$, defined as

5 $$\begin{aligned} {\bar{v}}_i'=\max \left\{ 1-\gamma \sigma ,0\right\} \cdot {\bar{v}}_i. \end{aligned}$$

Notice that a sudden change in the participant *HR* value would determine an equally sudden variation of the value of ${\bar{v}}_i'$, thus leading to a prompt reaction from the robot side (indeed, the consequent variation of the actual robot speed would typically take place in less than a second). A value of the constant coefficient $\gamma$ that is too small would lead to little difference between the original version of the algorithms and their modified versions. On the other hand, a value of $\gamma$ that is too high would drastically reduce cobot speed even for small increases of HR beyond $HR_0$, thus hindering productivity. To strike the balance between these two extreme cases, we decided to set $\gamma =0.5$ via trial and error.

### Hypotheses

The following hypotheses were tested in our experiments:

**H1**: a. cobot productivity with MPC is higher than with FP;
  b. cobot productivity with MPC-HR is higher than with FP-HR;
  c. cobot productivity with FP is higher than with FP-HR;
  d. cobot productivity with MPC is higher than with MPC-HR;
**H2**: a. perceived safety with FP is higher than with MPC;
  b. perceived safety with FP-HR is higher than with MPC-HR;
  c. perceived safety with FP-HR is higher than with FP;
  d. perceived safety with MPC-HR is higher than with MPC;
**H3**: perceived safety increases with time within the same experimental session due to habituation;
**H4**: perceived safety increases proportionally with previous participants’ experience interacting with robots.

**Figure 2:**
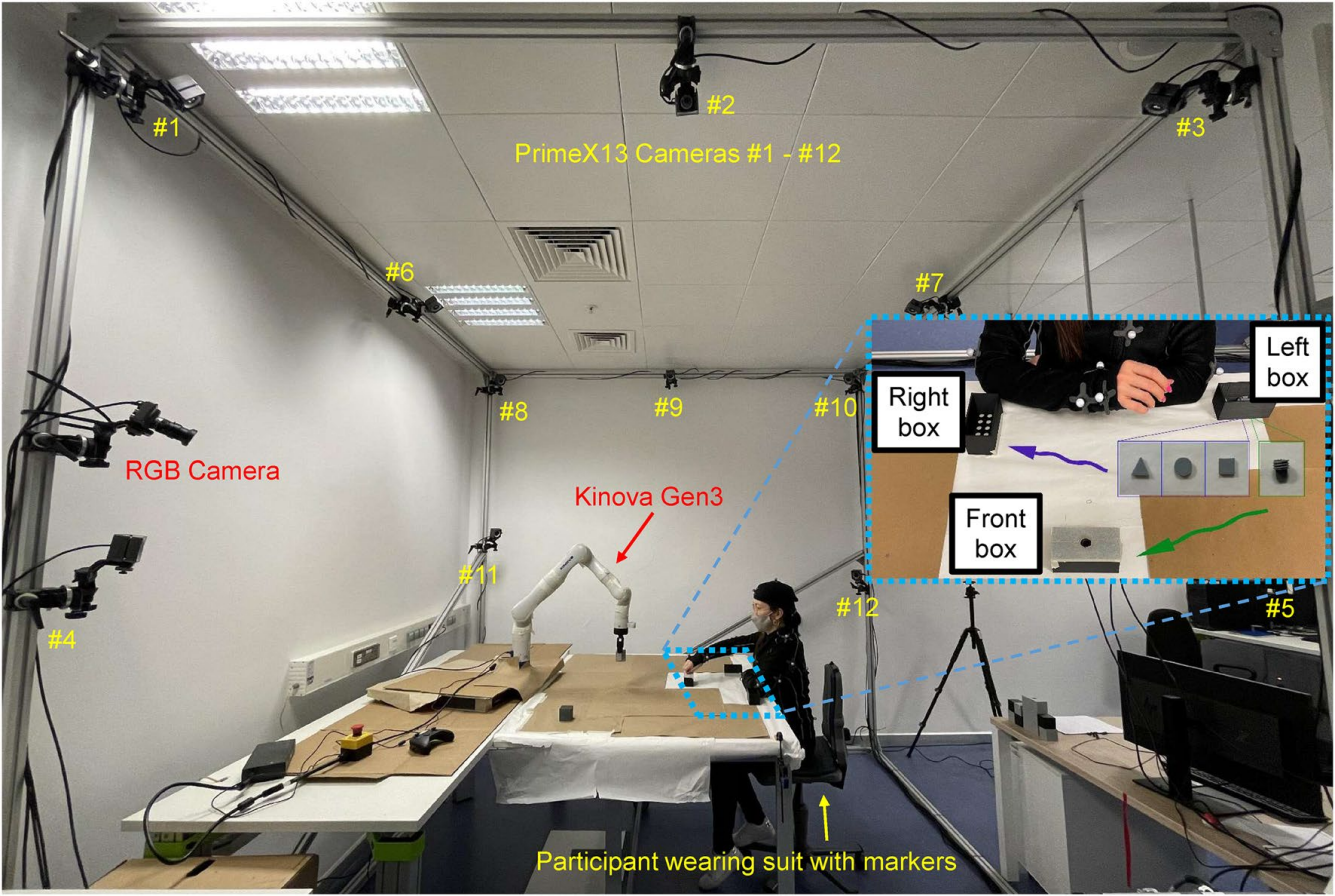
Experimental setup. In this figure one can see the 12 PrimeX13 cameras and the RGB camera that compose the OptiTrack motion capture system, together with the supporting cube frame. Inside the cube one can see the table in which the Kinova Gen3 cobot is placed, and on which the human participant, wearing the suit with markers, carries out the given task. A detailed view of the task is shown in the box in the right part of the figure. Similarly to Fig. 1, the shown human operator is an author of this paper.

### Experimental setup

The experimental setup is shown in Fig. 2. The employed cobot was a Kinova Gen3, an ultra-lightweight robotic arm explicitly designed for pHRI. The cobot was connected to a Linux PC with Intel Core i9-7900X CPU and 16GB RAM, running Robot Operating System (ROS), on which the motion planning algorithms were implemented. The MPC and MPC-HR algorithms were run in real time through C code generated via the software toolbox ACADO^36^ (version 1.2.1beta, available at https://acado.github.io/) using a sampling interval of 50 ms; the implementation details in terms of employed optimization algorithms and related details are unchanged compared to the MPC algorithm described by Oleinikov et al.^12^, and thus are not reported in detail here.

The human motion was detected by an OptiTrack system, composed of one RGB camera and 12 OptiTrack PrimeX13 cameras. The cameras were mounted on a supporting frame constructed using aluminium extrusions, which were connected together to form a rigid cube frame. The OptiTrack system was interfaced with the Motive software, running on a dedicated Windows PC (also with Intel Core i9-7900X CPU and 16GB RAM). The motion capture data were streamed from the Windows PC and received by the ROS machine via NatNet Client. As the OptiTrack system was of marker-based type, each human subject had to wear a set of markers—attached to a special suit—whose locations were detected by the cameras in real time. The motion capture system was calibrated, with reference to the robot coordinate system, with a maximum error—equal to the above-mentioned measurement precision $\varepsilon _s$—of 4 mm and a maximum time delay of 11 ms, composed of acquisition period, transition delay and software delay.

The HR data was instead acquired by the above-mentioned Empatica E4 wristband, which was connected to a mobile phone app (“E4 realtime”) via Bluetooth. The E4 realtime app was connected to the ROS machine via TCP/IP protocol, and provided an updated HR value every second. The wristband includes a photoplethysmography sensor that measures blood volume pulse, from which it derives the inter beat interval (IBI). An inbuilt algorithm in the wristband directly removes wrong IBIs^37^. In our experiments, the resulting HR value is also low-pass filtered using a three-sample moving average filter, to reduce the possible effect of measurement noise.

### Experimental procedure

A total of 48 healthy human subjects participated in the experiments. They were all either students—either undergraduate or graduate students in robotics, psychology, education and chemistry—or employees—laboratory technicians and managers, and administrative employees—of Nazarbayev University, with an age range between 18 and 38 years old. Each participant was involved in a single session, with duration of about 30 minutes. Before starting the experimental session, each participant read and signed the provided consent form and answered a pre-experiment questionnaire, which contained questions on age, attitude towards sharing their physical space with robots, and previous experience working with robots. After that, a video was shown to the participant showing the task to be executed. The participant would then proceed wearing the OptiTrack suit with markers and the E4 wristband on the left wrist, and would sit on the chair shown in Fig. 2. The next activity consisted of running the task previously explained in the video, and also depicted in Fig. 2 (in the box in the right side of the figure), with the cobot standing still. The task was executed with the right hand, while keeping the left arm in the rest position shown in the box in Fig. 2, and consisted of picking up one out of four types of small elements (screw, triangle, square, circle) from a box located on the left of the participant, and either inserting it in the box located to the right (for triangles, squares and circles), or screwing it into the box located in front of the participant (for screws). This description of the task is valid for right-handed subjects, while, for left-handed participants, the words “left” and “right” in the description have to be swapped. The task was repeated for a duration of 4 minutes, and the average HR value acquired during task execution was set as $HR_0$. After a short break, the subject would execute the same task for 4 minutes while the robot executed its pick-and-place task using one of the four described motion planning algorithms, moving cubes from the left to the right—and vice-versa—of the participant. At the end of the 4 minutes slot, the subject would fill a questionnaire about perceived safety during the experiment, whose questions are explained in detail in “Measures” in the following. This activity would last for about one minute, and during this time a new value of $HR_0$ would be determined for the next experiment, in order to adjust it to the fact that executing the task might lead to a slight HR increase, which cannot be attributed to stress, but just to physical activity. Then another experiment with another algorithm would follow with a corresponding questionnaire, running in total all four algorithms. The order of the motion planning algorithms was changed, so that each of the possible sequences was experienced by exactly two participants (there are 24 permutations for a set of 4 elements), to minimize the influence of habituation on the assessment of perceived safety for each algorithm. At the end of the last questionnaire, the participant was also asked to rank the four algorithms—about which no details were given—from the safest to the least safe. A video of the experiments for one of the subjects and all four motion planning algorithms is provided in the Supplementary Material, together with the corresponding time evolution of relevant variables.

The described experimental procedure followed for all 48 subjects was approved by the Nazarbayev University Institutional Research Ethics Committee (NU-IREC), which follows the principles set out in the report “Ethical Principles and Guidelines for the Protection of Human Subjects of Research”, also known as “Belmont Report”. More in detail, an application titled “Experiments on human–robot workspace sharing” was submitted to NU-IREC, and approval was obtained on June 20, 2021. Informed consent was obtained in writing from all participants. A separate informed consent was obtained, when relevant, to publish information, photographs and/or videos in an online open access publication.

### Measures

In order to test the formulated hypotheses, the following independent and dependent variables were taken into account. The independent variables were:

- The *motion planning algorithm* and the *order of execution* of each algorithm within each experiment, both decided by the researcher who was running the experiment, and thus both perfectly known.
- The *previous experience* of the participant, obtained in the pre-experiment questionnaire as an answer to the question: “Have you ever worked/interacted with a robot?” The possible answers were (1) “never”, (2) “once or twice”, (3) “often”, or (4) “I work with robots”.

The dependent variables were:

- The *cobot productivity*, indirectly assessed by the average task completion time (ATCP). This is defined as the mean value of the time interval—within a 4-minutes time slot—that the cobot needed to pick a cube from its resting position and place it at its goal position. As the distance between these locations remained approximately constant, the ATCP value only depended on the employed algorithm and on the human motion. A larger ATCP implies fewer completed pick-and-place tasks in the same time interval, and therefore the ATCP is inversely proportional to productivity.
- Two 5-point Likert scales for agreement in the pre-experiment questionnaire—“I would feel nervous while interacting with the robot (1-strongly disagree to 5-strongly agree)” and “I would feel nervous while sitting in front of the robot (1-strongly disagree to 5-strongly agree)”—which were merged by averaging the rates, and the obtained scale was named *pre-experiment nervousness*. These questionnaire items were obtained via slight modifications of the questions in the Negative Attitude Towards Robots (NARS) questionnaire^38^. More precisely, the first question is a modification of NARS item 8 (“I would feel nervous operating a robot in front of other people”), modified to account for the fact that the human subject in our experiments did not operate the robot, but rather physically interacted with it; the second question is a modified version of NARS item 10 (“I would feel very nervous just standing in front of a robot”), changed considering that our participants would be sitting rather than standing.
- The *perceived safety* experienced by the participant. This was evaluated by five different measures (two physiological and three subjective), and precisely:
  1. The *average HR* value during the 4-minutes time slot. For the same participant, a higher *average HR* value is an indicator of higher physiological stress^18^, which indicates lower perceived safety.
  2. The *average* $\sigma$ during the 4-minutes time slot. This indicator is clearly related to the previous one, but there is no 1-to-1 correspondence between the two.
  3. Two 5-point Likert scales for agreement in the post-experiment questionnaire - “I felt nervous while interacting with the robot (1-strongly disagree to 5-strongly agree)” and “I felt nervous while sitting in front of the robot (1-strongly disagree to 5-strongly agree)” - merged by averaging the rates (Cronbach’s $\alpha =.87$)^39^, thus obtaining a single scale named *nervousness*. This is the post-experiment version of the *pre-experiment nervousness* scale described above.
  4. Two 5-point semantic differential scales to answer to the requests “Please rate your emotional state on this scale” from “anxious” to “relaxed” and from “calm” to “stressed” were merged by averaging the rates (Cronbach’s $\alpha =.844$). These represent a subset of questions of the Godspeed questionnaire on perceived safety^22^, in which the original word “agitated” was substituted with “stressed”, as we assumed that this word could be more easily understood by the participants, who were all non-native English speakers. The obtained differential scale (DS) was simply named *perceived safety DS*.
  5. The *ranking* of the experienced cobot motions, as answer to the question “Sort the four experiments (1-4) from the one in which you felt the safest to the one in which you felt the least safe”, asked to the participant at the end of the whole experimental session.

## Results

A series of Kolmogorov–Smirnov (K–S) and Shapiro–Wilk tests^40^ were conducted on all dependent variables, overall and within groups, to test the assumption of normality. We mainly focus on reporting significant differences (identified by a *p*-value satisfying $p<0.05$), due to space limitation; however, the data obtained for each of the 48 participants are available as supplementary material online in Tables S1 and S2. For each described dataset, we report information on center and variability as (mean ± standard deviation).

### Cobot productivity

**Figure 3:**
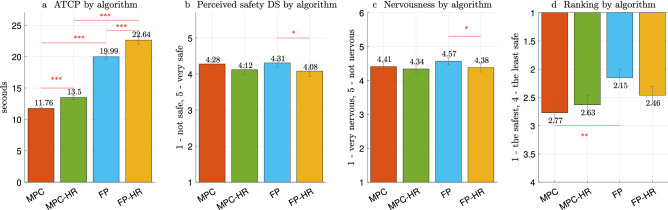
Mean values for different motion planning algorithms: (**a**) ATCP (inversely proportional to *cobot productivity*); (**b**) *perceived safety DS*; (**c**) *nervousness*; (**d**) *ranking*. Significance levels for pairwise comparisons are indicated as * for $p< 0.05$, ** for $p<0.01$, and *** for $p< 0.001$. The error bars display standard deviation.

In order to check H1, a Friedman test^41^ was applied to ATCP values (inversely proportional to productivity). There was a statistically significant difference in *cobot productivity* between algorithm types: $\chi ^2 (3)=127.575$, $p < 0.001$. As presented in Fig. 3a, the cobot was significantly more productive with MPC ($11.76\pm 0.78$) in comparison to FP ($19.99\pm 2.87$): $\chi ^2 (1)=48.000$, $p < 0.001$, while, with MPC-HR ($13.50 \pm 2.10$), the cobot was significantly more productive in comparison to FP-HR ($22.64 \pm 4.61$): $\chi ^2 (1)=48.000$, $p < 0.001$. Therefore, we can accept H1a and H1b.

Similarly, productivity with FP ($19.99 \pm 2.87$) was also significantly better in comparison to productivity with FP-HR ($22.64 \pm 4.61$) ($\chi ^2 (1)=16.333$, $p < 0.001$) and productivity with MPC ($11.76 \pm 0.78$) was also significantly better in comparison to productivity with MPC-HR ($13.50 \pm 2.10$) ($\chi ^2 (1)=$30.083, $p < 0.001$). Therefore, we can accept H1c and H1d.

### Perceived safety

In order to test H2, a series of Friedman tests was conducted on the *perceived safety* metrics indicated in Measures. For the physiological metrics (*average HR* and *average* $\sigma$ values), we did not find significant differences between algorithms.

There was a statistically significant difference in participants’ ratings of *perceived safety DS* between FP and FP-HR: $\chi ^2 (1)= 4.172$, $p = 0.041$. As shown in Fig. 3b, participants rated their *perceived safety DS* significantly higher for the FP algorithm ($4.31 \pm 0.83$) compared to FP-HR ($4.08 \pm 1.05$).

Next, a series of Friedman tests was also applied to the *nervousness* metric. There was a statistically significant difference in the *nervousness* scale between FP and FP-HR: $\chi ^2 (1)= 3.857$, $p = 0.049$. Similarly to *perceived safety DS*, we found a significant difference between the two FP-based algorithms—with or without HR-based speed modulation—since FP ($4.57 \pm 0.78$) was rated as making the participants less nervous than FP-HR ($4.38 \pm 0.90$), as presented in Fig. 3c.

Finally, a Friedman test resulted in significant differences in *ranking* (Fig. 3d) between MPC and FP: $\chi ^2 (1)= 8.333$, $p = .004$. FP was ranked as the algorithm generating the safest robot motion ($2.15 \pm 1.01$), while MPC was ranked as the least safe algorithm ($2.77 \pm 1.12$).

To conclude, we can partially accept H2a as confirmed by the *ranking* metric, and reject H2b-d.

### Effect of habituation

In order to check H3 we analyzed the *perceived safety* distribution depending on the order in which each of the consecutive cobot motions (executed for 4 minutes each and with different algorithms in all possible sequences) were run, independently from the employed algorithm.

**Figure 4:**
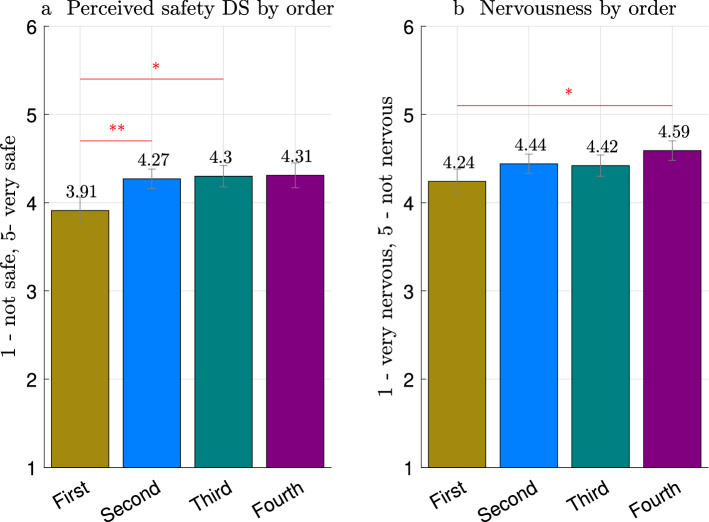
Mean values based on the order of execution: (**a**) *perceived safety DS*; (**b**) *nervousness*. Significance levels for pairwise comparisons are indicated as * for $p<0.05$, ** for $p< 0.01$, and *** for $p< 0.001$. The error bars display standard deviation.

A Friedman test resulted in a statistically significant difference in *perceived safety DS* depending on the order of execution: $\chi ^2 (3)= 9.572$, $p = 0.023$. There was a statistically significant difference between the first and second cobot motions: $\chi ^2 (1)= 10.800$, $p = 0.001$, and between the first and third motions: $\chi ^2 (1)= 4.235$, $p = 0.040$, as shown in Fig. 4a. The first cobot motion had significantly lower rating ($3.91 \pm 0.15$) of *perceived safety DS* compared to the second ($4.27 \pm 0.11$) and third ($4.30 \pm 0.12$) motions.

Next, a series of Friedman tests was also applied to the nervousness metric. There was a statistically significant difference in the nervousness scale between the first and fourth cobot motions: $\chi ^2 (1)=3.846$, $p= 0.049$. Participants felt significantly more nervous with first ($4.24 \pm 0.14$) than with fourth ($4.58 \pm 0.11$) cobot motions (Fig. 4b).

To conclude, we can accept H3 as confirmed by subjective metrics including *perceived safety DS* and *nervousness*.

### Previous Experience with Robots

For the next analysis, we categorized participants in two groups, based on their *previous experience*: inexperienced (those who, to the question “Have you ever worked/interacted with a robot?”, responded “never” or “once or twice”) and experienced (those who responded “often” or “I work with robots” to the same question). As a result, 34 participants were labeled as inexperienced and 14 as experienced. There was no statistically significant difference between groups both by algorithms and by order of execution, concluding that H4 is rejected.

## Discussion

Our results fully support H1. However, as for H1a and H1b, we would like to remark that the superior performance of MPC over FP (and of MPC-HR over FP-HR) would not be necessarily observed for any type of human motion. Indeed, replanning the cobot trajectory based on the current human location is convenient only if the human obstructs the cobot motion for relatively long periods of time (i.e., a few seconds, as in our experiments). If this obstruction happens for very short time intervals, then replanning the cobot motion on a longer path (as MPC and MPC-HR do) would be less convenient than simply waiting for a short time and then proceed along the most convenient path in the absence of humans (as FP and FP-HR do). On the other hand, regarding H1c and H1d, as the cobot speed decreases in MPC-HR with respect to MPC and in FP-HR with respect to FP, we would expect that the productivity of the HR-based algorithms would never be higher than the one of those without HR-based speed reduction, and therefore we expect H1c and H1d to be valid for any human motions.

Additionally, one of our subjective metrics (ranking) allows us to accept H2a, suggesting that *perceived safety* was higher in FP in comparison to MPC. When running FP, the cobot moved along a fixed path by definition, and with a lower velocity (both features typically being associated to higher perceived safety in the robotics literature^42–46^), whereas in the MPC case the cobot at times moved faster and was in general more unpredictable. This conclusion is therefore in line with previous findings in the literature. Although H2b-d were rejected, we found an opposite trend in the relationship between the *perceived safety* assessments in FP and FP-HR. Indeed, both *perceived safety DS* and *nervousness* show that FP was perceived in general as safer than FP-HR. This might be due to the fact that the average lower speed of the cobot when running FP-HR also led to more frequent stops, thus making the human participants more nervous and anxious.

An important conclusion that merges the findings on H1 and H2 is that, at least based on our experiments, introducing the HR-based speed reduction in either FP or MPC does not lead to any advantages, as it decreases productivity and does not affect perceived safety (or, for FP, it worsens it). As mentioned above, the decrease of productivity is largely expected, as employing HR-based methods reduces robot speed. However, the lack of improvement in perceived safety is unexpected, and it is thus natural to wonder if this result is in contrast with previous findings in the literature. As already mentioned in the introduction, few works have been published in which the behavior of a robot was modified in real time to respond to human physiological feedback^24–27^. Of these, only the work of Kulic and Croft^27^ deals with the interaction between a human and a manipulator, and is thus the only paper whose results can be compared with ours. In it, the robot motion was planned at three different levels (long, medium and short term), and only the medium-term and short-term planners were influenced by physiological feedback. In particular, the so-called danger index, which influenced medium and short-term planning, was calculated based on the location and speeds of robot and user, but also on the user head orientation and on the physiological measurement of the affective state, reducing the robot speed proportionally to the detected level of anxiety. In their experimental results, Kulic and Croft^27^ showed the effects of this further speed reduction in terms of robot motion, but they did not evaluate how introducing this element in the motion planning algorithms affected the participant’s perception of safety. As a matter of fact, reducing the robot speed when the level of anxiety increases seems an obvious way of reducing anxiety. In Kulic and Croft’s case, the robot was an industrial manipulator, and its motion was not planned based on SSM, which was introduced several years later. Therefore, we would expect that, in their case, an evaluation of human anxiety would have shown an improvement when physiological feedback was used to reduce robot speed. In the case described in our paper, however, the manipulator is a cobot (much lighter than industrial manipulators), and the motion satisfies SSM ; as a consequence, participants were probably already feeling very safe when no further HR-based speed modulation was introduced, and this is probably the reason why a further speed reduction did not influenced perceived safety.

The effect of habituation was demonstrated in our findings, based on subjective metrics, which support H3. This is in line with results in the robotics literature^42,47–49^, as participants typically get accustomed to the experimental site and to the robot motion.

Although previous works^38,50^ claimed that previous experience with robots would influence the safety perception of the participants, this was not confirmed in our study. The reason is probably that the cobot motion with all the devised algorithms is safe according to the SSM principle, and thus is also generally perceived as safe, due to the relatively low cobot speed, mass and size. This allows inexperienced participants to quickly get accustomed to physically interact with the system, thus strongly reducing the differences between experienced and inexperienced subjects.

Possible future directions of this work would be the extension of the range of participants to include industrial workers, and the perceived safety evaluation of newly-defined safe motion planning algorithms.

## Supplementary Information

Supplementary Information 1.

Supplementary Information 2.

## Data Availability

The dataset generated during the study described in this paper is available in the Supplementary Material in the form of Tables S1 and S2. A video of the experiments for all four algorithms for one participant is also available in the Supplementary Material. Additional data are available from the corresponding author on reasonable request.
